# Advancing Health Equity Through Equity-Centered Leadership Development with Interprofessional Healthcare Teams

**DOI:** 10.1007/s11606-022-07529-x

**Published:** 2022-06-03

**Authors:** Giselle Corbie, Kathleen Brandert, Cheryl C Noble, Ellison Henry, Gaurav Dave, Rachel Berthiume, Melissa Green, Claudia S P Fernandez

**Affiliations:** 1 Center for Health Equity Research, Department of Social Medicine, School of Medicine, University of North Carolina at Chapel Hill, Chapel Hill, NC USA; 2 Department of Medicine, School of Medicine, University of North Carolina at Chapel Hill, Chapel Hill, NC USA; 3 Office of Public Health Practice and Department of Health Promotion, Social and Behavioral Health, College of Public Health, University of Nebraska Medical Center, Omaha, NE USA; 4 Leadership Evaluation Consultant, Scotts Valley, CA USA; 5 Department of Maternal and Child Health, UNC Gillings School of Global Public Health, University of North Carolina at Chapel Hill, Chapel Hill, NC USA

**Keywords:** leadership development, equity-centered leadership, health equity

## Abstract

**Introduction:**

Events of spring 2020—the COVID19 pandemic and re-birth of a social justice movement—have thrown disparities in disease risk, morbidity, and mortality in sharp relief. In response, healthcare organizations have shifted attentions and resources towards equity, diversity, and inclusion (EDI) issues and initiatives like never before. Focused, proven equity-centered skill and mindset development is needed for healthcare professionals to operationalize these pledges and stated aims.

**Aim:**

This article highlights program evaluation results for this Clinical Scholars National Leadership Institute (CSNLI) specific to EDI. We will show that CSNLI imparts the valuable and essential skills to health professionals that are needed to realize health equity through organizational and system change.

**Setting:**

Initial cohort of 29 participants in CSNLI, engaging in the program over 3 years through in-person and distance-based learning offerings and activities.

**Program Description:**

The CSNLI is a 3-year, intensive leadership program that centers EDI skill development across personal, interpersonal, organizational, and systems domains through its design, competencies, and curriculum.

**Program Evaluation:**

A robust evaluation following the Kirkpatrick Model offers analysis of four data collecting activities related to program participants’ EDI learning, behavioral change, and results.

**Discussion:**

Over the course of the program, participants made significant gains in competencies related to equity, diversity, and inclusion. Furthermore, participants demonstrated growth in behavior change and leadership activities in the areas of organizational and system change. Results demonstrate the need to center both leader and leadership development on equity, diversity, and inclusion curriculum to make real change in the US Healthcare System.

**Supplementary Information:**

The online version contains supplementary material available at 10.1007/s11606-022-07529-x.

## INTRODUCTION

While health inequities have long been recognized, events of 2020—the COVID19 pandemic and re-birth of a social justice movement—have thrown disparities in disease risk, morbidity, and mortality in sharp relief. From the COVID mortality rates in black and brown population to the designation of high-risk front-line “essential jobs” commonly filled by underrepresented minorities and immigrants, current health, social, and economic events have exposed the racism that permeates our country’s systems. Persistent and pervasive health inequities in the United States (US) begin at birth—starting with which babies are more likely to be carried to term and celebrate their first birthday^1–4^—and compound throughout a person’s life. Inequities, rooted in structural racism through historical policies and practices, are continued through current-day norms of our social and health systems and institutions, and are perpetuated by the myth that responsibility for an individual’s health outcomes lies solely within that individual’s sphere of control. The design and approaches of patient care in the American healthcare system further reinforce that myth. While the healthcare workforce still has the trust of the communities they serve, there is a particular responsibility and opportunity for that workforce to address inequities.

In the wake of spring 2020, healthcare professional organizations and health professional schools have drafted and posted equity pledges noting the roots of structural racism, espousing the need for diversity, and committing to social justice. New positions for chief diversity officers have been created. Curriculum committees raced to find ways to include diversity, equity, and inclusion topics in undergraduate and graduate medical education. New committees have been formed to address diversity and inclusion at institutions. These changes are often championed by a small group of people who already recognize the need for change, leveraging new organizational enthausiam. However, for durable institutional shifts to occur, we need leaders throughout health systems and institutions with the knowledge, attitudes, and skills to dismantle structures that maintain health inequity and establish new structures that promote health equity.

This paper describes our findings from the first cohort of an interprofessional leadership development program that uses an equity-centered approach to drive curricular components. We describe program evaluation results which illustrate how curricular competencies translate into participant learning, behavioral change, and ultimately organizational and community impact to advance health equity.

## METHODS

### Program Description

In 2015, the Robert Wood Johnson Foundation funded the Clinical Scholars (CS) National Leadership Institute, a leadership development program for clinicians that weaves the concepts of leadership, equity, diversity, and inclusion together in a robust 3-year curriculum. The CS mission is to “develop adaptive leaders from all health disciplines to extend their influence and impact through transformative leadership training centered in equity, diversity, and inclusion.” CS uses an equity-centered approach to teach leadership strategies to healthcare professionals, imparting skills to impact health inequities in their communities and organizations.^5^ Interprofessional teams apply to CS with a proposed Wicked Problem Impact Project (WPIP) that serves as their action learning project during the program. The goal of the WPIP is to generate positive community impacts by advancing health equity, promote sustainability, and to the extent possible, achieve scalability. Enrolled teams focus on a wide variety of health equity issues, from mental and physical health to healthcare deserts to community systems that support health. All projects are listed at the CSNLI website (ClinicalScholarsNLI.org). CS supports successful WPIP implementation by fostering and strengthening participants’ skills as teams and as individuals.

#### Competencies

**Figure 1:**
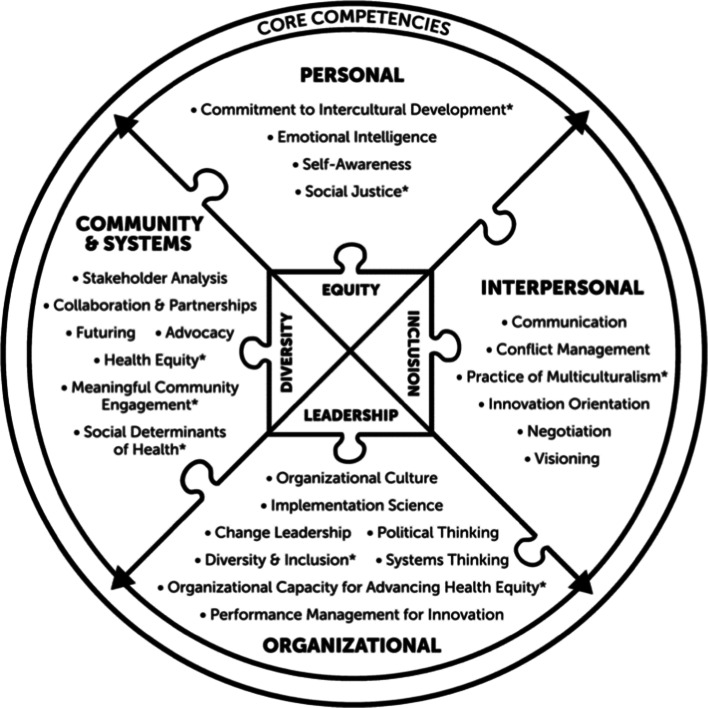
**Core competencies and competency domains of clinical scholars program**

Twenty-five leadership competencies stand at the core of CS’s equity-centered framework. The competencies merge traditional leadership proficiency (e.g., self-awareness, negotiation skills, policy and advocacy skills) with skill sets in the areas of equity, diversity, and inclusion (EDI) (e.g., intercultural development and community engagement). The competencies are grouped into four domains: *Personal*, *Interpersonal*, *Organizational*, and *Community & Systems* (see Fig. 1). As a set, these competencies encompass the concepts needed to internally reckon with and externally live out equity-centered leadership. Figure 1 indicates which competencies are directly related to EDI skill sets with an asterisk. Linkages across all of the 25 competencies have been intentionally made to promote the interconnectivity that centers the leadership training on equity.

#### Curriculum

Over the course of the 3-year program, participants are challenged to learn, try on, and then fully step into the mindsets that help them grow from being individual contributors into being leaders of teams, communities, and systems. Sessions, activities, self-reflections, and assignments create opportunities in which participants learn, practice, and apply the skills, behaviors, and attitudes. Formats vary and include didactic lectures, small- and large-group discussions, case study debriefs, practice scenarios, book studies, reflective writing activities and simulation experiences. The leadership and EDI curriculum components exist both in tandem and in unison to provide the full CS experience, at times interweaving fully. A more detailed description of the pedagogical theory and curricular details can be found elsewhere.^6^

#### Evaluation Conceptual Framework

Social cognitive theory,^7,8^ the socioecological model,^9,10^ and the social capital framework^11,12^ guide CS evalulation. Together, these theories suggest that an individual (and therefore their behaviors, decisions, and learnings) is influenced by their interpersonal relationships, environment, community, professional network, and broader society. Our evaluation design recognizes and embraces this complexity and uses Kirkpatrick’s Four-Level Training Evaluation model ^13–15^ to frame implementation of a mixed-methods and multilevel evaluation approach. A detailed description of the CS evaluation approach is presented elsewhere.^16^ For the purposes of this paper, we explore Kirkpatrick's model levels 2-4, as defined below:

- Level 2: Learning—participants’ gains in knowledge, self-efficacy, skills, and attitudes of EDI-focused competencies
- Level 3: Behavior—translation of gained knowledge and skills into tangible EDI-relevant actions
- Level 4: Results—EDI-relevant impacts on participants’ individual leadership, organizations, and communities

### Data Collection and Analysis

Aligned with our evaluation framework, we describe four data collection activities and the related methods for analysis used to evaluate CS EDI learning, behavior, and results.

#### Level 2: Competency Assessment

To assess change in eight EDI competencies, we used a retrospective pre/post-test design—an approach demonstrated to reduce biases commonly associated with self-report data collection. ^17–19^ In a more traditional “pre-test, training, post-test” evaluation design, there is concern of a confounding response shift bias when participants self-rate items at two separate timepoints. Response shift bias is when a participant’s internal frame of reference for the item being measured changes, likely as a result of the training they attended where they learned about said item. Evaluative comparisons of the two designs have shown that a retrospective pre-test method can be used to control for the response shift bias. This design asks the participant to self-rate items at a *single* timepoint but reflect back on the “pre-training” time.

We collected data at three timepoints (baseline: 0–6 months, mid-point: 12–18 months, endpoint: 30–36 months). At each timepoint, we asked participants to rate an item for each dimension for each competency on a 7-point Likert scale, with one being the lowest level of agreement with the item and seven being the highest level of agreement, based on their levels 6 months prior and now (*see example below*). Participants provided two ratings for each item—one rating for 6 months prior (retrospective pre-rating) and for current day (for example, Please rate your level of knowledge of self-awareness 6 MONTHS AGO- 1: none through 7: expert; Please rate your level of knowledge of self-awareness NOW- 1: none through 7: expert). We assessed four dimensions of each competency—knowledge, attitude, self-efficacy, and intention to use across the four competency domains.

We performed descriptive statistics at each timepoint using SPSS^20^, creating composite variables using participants’ reported levels of knowledge, self-efficacy, attitude, and intent to use each of the 25 leadership development competencies (Appendix 1). From the composite data, we analyzed participants’ growth along the evaluative dimensions and competency domains. In the final phase of analysis, we ran Wilcoxon’s signed-rank tests using baseline (0 months) and endpoint (36 months) composite ratings for each individual competency, since competency rating data were not normally distributed and sample sizes of participants were small.

#### Level 3: Behavioral Examples

Participants completed behavioral statements describing how they used each of the 25 CS competencies in their work during their time in CS as Online Wisdom Logs (OWLs). Submissions via Canvas Learning Management System^21^ used the STAR (Situation, Task, Actions, and Results) rubric.^22^

We used an inductive approach in initial analysis of OWLs to identify common themes of how participants reported using each of the eight EDI competencies. An evaluation staff member read through the statements submitted for each EDI competency and summarized the type of use described (e.g., advocacy for more diverse membership in key stakeholder groups). Use types were categorized into thematic groups, (e.g., advocacy) for each competency. We then calculated frequencies to determine the number of examples within each competency.

#### Level 4: Most Significant Change

The Most Significant Change (MSC) evaluation approach to assess training outcomes^23^ is a participatory qualitative process that collects participants’ stories describing their most significant change during or resulting from participation in a program. MSC provides detailed data about how participants use the training concepts and competencies in their personal and professional lives, thus providing a more nuanced understanding of program outcomes. As part of their final report of WPIP project activities, each participant was asked to submit a story of what they felt was the most significant change they experienced during the program by responding to the following prompt:

Describe in one or two paragraphs the most significant change that has resulted from your involvement with the Change Leadership Initiative. Please describe the situation, task, actions, results, or other details you can relate to the change.

Stories were coded in three rounds and incorporated thematic and phenomenological analysis methodologies.^24–26^ An initial codebook based on CS goals grew as codes emerged as key themes from the first round of coding and were focused specifically on segments coded under “equity, diversity, inclusion.”^27^

#### Level 4: Tracking of Reported Leadership Activities

Data was collected regarding participants’ leadership activites via multiple strategies: direct communication from participants to program staff, targeted Google alerts, an online submission form and through annual review of CVs/resumes. Participants were encouraged throughout the 3-year program and at regular intervals after graduation to submit leadership activites. The types of activites collected include writing, training, and advocacy activities; achieving changes in public, organizational, and/or tribal policy; and formal recognition (e.g., awards, career advancement, and news coverage). Participants were also encouraged to report activites outside of the specific categories listed above. We report here activities tracked from September 2019 through April 2021.

A deductive analysis approach identified EDI themes present in submitted leadership activities. CS staff assigned an activity type (e.g., writing, award, training) to each item based on the selection made by the participant, or after reviewing the definition of each activity type, highlighting those that specifically discussed topics related to EDI competencies. We then calculated frequencies to determine the number of EDI activities per activity type.

## RESULTS

### Sample

The sample consists of 27 of the 29 participants in the first cohort of CS enrolled in the program from Fall 2016 to Fall 2019 (Table 1). All participants reported six or more years of practice in medicine and nursing (63%), followed by social work, psychology, dentistry, occupational therapy, and pharmacy. Participants represented seven US states and one US district.

**Table 1:** Demographic Characteristics of the First Cohort of Participants of the CS Program

Demographic Characteristic	Count(%)
Gender*^,†^	
*n*	29
Male	12 (41%)
Female	17 (59%)
Race*	
n	28^‡^
White/Caucasian	14 (48%)
Black/African American	7 (24%)
Asian	4 (14%)
American Indian/Alaska Native	1 (3%)
Native Hawaiian or Pacific Islander	0
Bi-racial or multi-racial	0
Other	2 (7%)
Ethnicity*	
n	29
Hispanic or Latinx	3 (10%)
Neither Hispanic nor Latinx	26 (90%)

*Obtained from self-report survey

^†^Gender options provided—Man, Woman, Transman, Transwoman, Gender queer/non-conforming, Other, prefer not to say

^‡^One participant declined to respond

### Level 2: Learning

#### Competency Assessment

**Figure 2:**
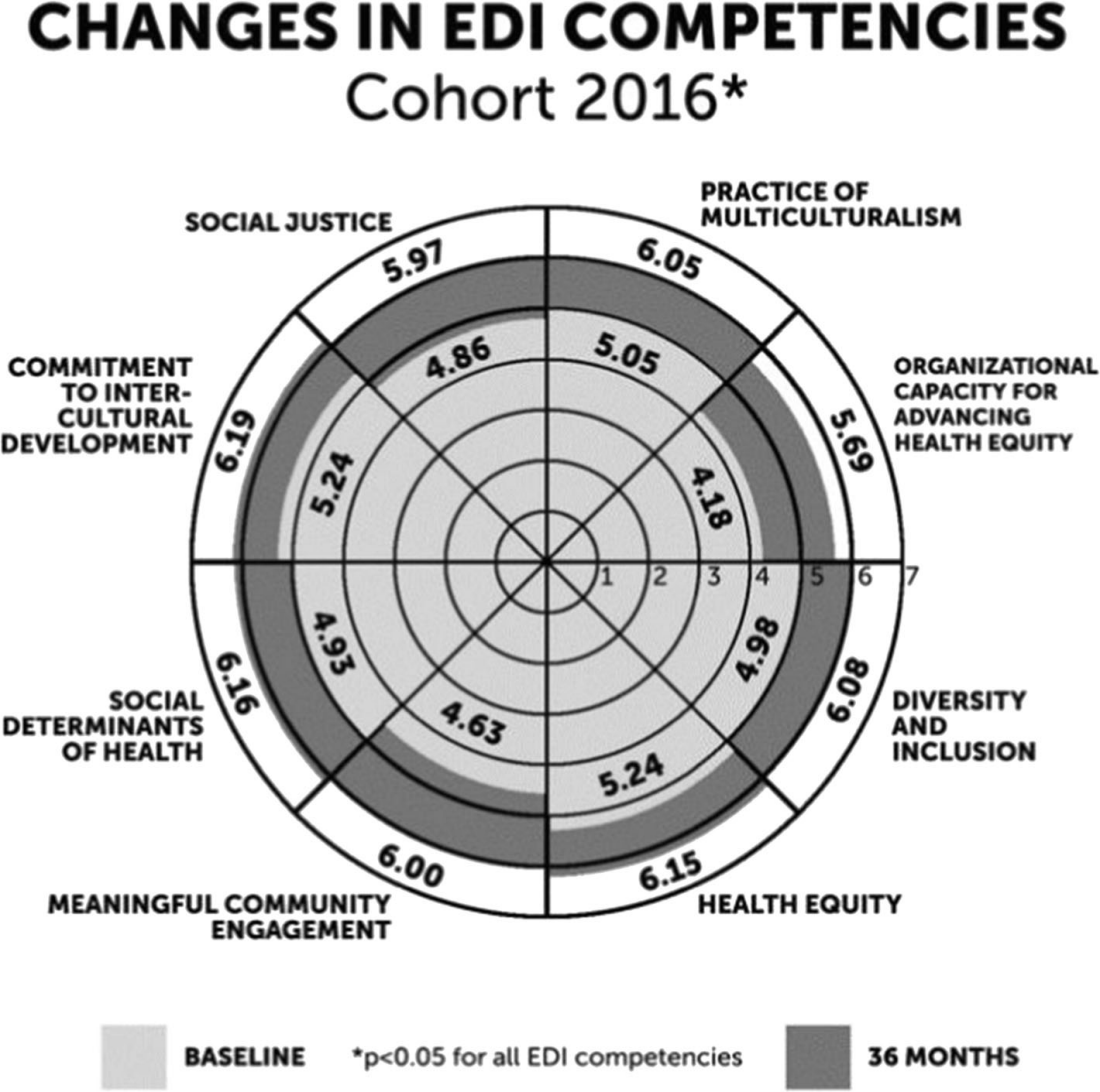
**Self-Reported changes in EDI competencies of CS Cohort 2016 Participants**

The radar chart (Fig. 2*)* shows changes in each EDI competency measured at the beginning (baseline) and end of CS. Each of the seven rings in the radar chart represents a value on the Likert scale of response options. The light gray shading indicates the average rating of a particular competency among Cohort 2016 Fellows at baseline. The dark gray shading indicates the average rating of a particular competency at endpoint. This graphic provides a visualization of the size of growth in each competency from baseline to endpoint. Endpoint competency scores were significantly higher than baseline (see Table 2 and Appendix 1) with the greatest change seen in “Organizational Capacity for Health Equity” (1.41 mean difference) followed by “Meaningful Community Engagement” (1.24 mean difference) (Table 2). Statistically significant gains occurred across all domains with participants reporting the most growth in the Organizational domain (1.23 mean difference).

**Table 2:** Self-Reported Changes in EDI Competencies of CS Participants from Baseline to 18 Months and 36 Months

Competency	*N*	Baseline mean (0 mo)	Mid-point mean (18 mo)	Endpoint mean (36 mo)	Difference in baseline and endline means (*^,†,‡^)	Standard deviation
Personal domain						
Commitment to intercultural development	26	5.24	6.06	6.19	0.87^†^	1.36
Social Justice	26	4.86	5.86	5.97	1.13^†^	1.55
Interpersonal domain						
Practice of multi-culturalism	26	5.05	6.01	6.05	0.93^†^	1.23
Organizational doman						
Organizational capacity for advancing health equity	24	4.18	5.51	5.69	1.41^†^	1.46
Diversity and inclusion	25	4.98	5.97	6.08	1.04^†^	1.43
Community and systems domain						
Health equity	26	5.24	6.07	6.15	0.86^†^	1.19
Meaningful community engagement	26	4.63	5.80	6.00	1.24^†^	1.56
Social determinants of health	26	4.93	6.08	6.16	1.17^†^	1.82

*p<.05

^†^*p*<.01

^‡^*p*<.001

Note: Difference in means calculated with pairs from Wilcoxon signed-rank tests. Means calculated from data set

Missing data excluded individually in mean calculation, excluded by pair in *t*-test calculation

### Level 3: Behavior

#### Behavioral Examples—Online Wisdom Logs (OWLs)

During the program, participants submitted 140 unique OWL statements describing use of eight EDI competencies. While participants used these competencies to achieve outcomes in multiple ways, the top two themes and examples for each competency are presented in Table 3. Across all the EDI competencies, participants described ways in which they used the competency to advance EDI in the projects or organizations in which they work. “Training Others” emerged as a theme across all competencies. The theme of Advocacy emerged within each of the eight different EDI competencies, with at least two participants reporting using that competency to advocate for EDI issues.

**Table 3:** Themes and Examples from CS Participants’ Reported Use of CS Competencies by Domain

Theme *(competency)*	Frequency of theme	Example
**Personal domain**		
*Commitment to diversity and social justice (n = 24)*		
Advocating	8	Participant advocated for the inclusion of a representative from a marginalized group in an institutional research advisory committee.
Developing new organizational systems or policies	6	Participant led the effort to develop an institutional policy to hire more staff and clinicians that reflect the racial and ethnic makeup of the client population.
*Social justice (n = 17)*		
Advocating	7	Participant facilitated the inclusion of parents of children with special healthcare needs to play a key teaching role in all training provided through their WPIP to healthcare professionals who provide medical and dental services to children with special healthcare needs, in order to advocate for the specific concerns of parents and children.
Using influence to promote social justice	4	Participant used their influence and connections to encourage and empower a client who is trans to advocate for themselves to be able to access the school locker room of their identified gender.
**Interpersonal domain**		
*Practice multiculturalism (n = 14)*		
Developing or updating program to be more culturally sensitive to clients	6	Participant ensured that client had access to traditional healer to support the client’s stated spiritual needs.
Taking personal steps to better understand cultures different than their own	4	Participant committed to learning about a client’s cultural background in order to provide the best medical care for that individual.
**Organizational domain**		
*Build organizational capacity to advance health equity (n = 17)*		
Facilitating expanded organizational and institutional capacity for EDI	6	Participant helped steer EDI Committee at their institution to move beyond data collection on EDI issues to actual implementation of programs and policies to expand diversity and inclusion.
Increasing access and/or services for marginalized communities	6	Participant initiated new program to increase insurance enrollment for an underserved population by ensuring materials are presented in clients’ language, providing translation, and developing culturally appropriate materials. These changes resulted in the clinic producing some of the highest insurance enrollment numbers of any clinics in their state.
*Foster diversity and inclusion (n = 18)*		
Advocating for increased diversity and inclusion in their organization/institution	6	Participant was invited to join their hospital system’s Diversity Committee. They advocated for policies that are more sensitive to the cultural and contextual needs of clients living in poverty.
Developing organizational policies	4	Participant played key role in updating their profession’s oath of practice for new clinicians to include “disability” as a factor that will not affect their judgement or ability to care for patients.
**Community and systems domain**		
*Health equity (n = 16)*		
Increasing access and/or services for marginalized communities	5	Participant helped facilitate a partnership between the university dental school and a local not-for-profit organization to provide dental services to children who are ineligible for state insurance due to immigration status and lack of citizenship.
Impacting organizational infrastructure	4	A participant led the creation of an Office of Community Initiatives at their hospital in order to support expanded screenings and services to address health inequalities in the community.
*Meaningful community engagement (n = 17)*		
Promoting community voices	10	Participant convened and led a focus group of transgender community members to inform the development of the website for a transgender health center.
Training others	2	Participant played a role in developing a workshop series where clients who are in addiction recovery to lead trainings for local community members on the causes, effects, and treatments for opiate dependency.
*Social determinants of health (n = 16)*		
Program improvements	4	In response to regular meetings with residents of a local community, a participant collaborated with the local hospital to develop a food pantry to address high levels of food insecurity present in the neighborhood.
Training others	4	Participant developed a class at their academic institution to focus on introducing students to the concepts of social determinants of health and how it will impact their work as future health providers.

### Level 4: Results

#### Most Significant Change (MSC)

Participants submitted 27 MSC stories with EDI a top theme identified in our analysis. Participants reported advocating for historically marginalized populations, teaching others, creating partnerships to address health equity issues, and expanding self-discovery around one’s own engagement with EDI (Table 4).

**Table 4:** Most Significant Change Findings Related to Equity, Diversity, and Inclusion

Theme	Frequency	Example
Advocating for marginalized groups	7	“We have worked extensively with the press to promote our center and vision to bring transgender healthcare from the margins to the mainstream.” “I have authored a chapter in a book on primary care for the transgender patient, and am presently writing an article on transgender medicine for the upcoming [publisher] encyclopedia of transgender studies.” “We are leveraging our [work from the program] for funding, growth planning, and a greater impact on transforming the culture of care for communities struggling with addiction, in [our state] and beyond.”
Creating programs that value EDI principles	6	“Eventually, a pilot program was developed that improved health equity for an impoverished community by providing clinical trauma screenings and behavior health services for [an urban] community in [state].” “Because of our...support from [the program], I am hopeful that the Gender Wellness Center will continue to thrive and be celebrated within our organization and our community.”
Exploring personal biases	4	“I have also appreciated the challenge and education around looking at bias. It is not always easy to do. … I have had the luxury of spending time talking about [bias] and how it affects different people. … That kind of time is a gift.”
Self-awareness/Personal learning around EDI principles	6	“I am now able to bring the best out of each person on my team in a way that I was never able to accomplish in the past.” “[In CS] I was exposed to societal analyses of gaps in health and social equity. I learned how to frame ideas and approaches to problems. I received guidance through the modules and books on how to navigate change and find my way to impact.” “I must advocate for health equity most notably for [people] who are at the highest risk. As an occupational therapist, I serve people who are living in poverty. I recognized that I do not fully understand their circumstances.”
Networking/Inten-tional relationship building	4	“Through relationships with stakeholders, most importantly the foster youth, and by recognizing the prevalence and ramifications of adverse childhood experiences in this special population, we have developed and implemented a wellness curriculum that helps youth manage the physiological changes their bodies have endured.” “[My new role as CEO] is a very exciting opportunity where I will be able to incorporate elements of our community mental health literacy program and create a new model of behavioral health wellness in this community.” “I used my social capital as a senior attending physician and president of my hospital’s medical staff to further advance [support of our LGBTQ+ initiative]. I met monthly with a Vice President, developing him as our champion within the organization. I also formed and am leading an Advisory Board to assure ongoing support and guidance from key leaders...within our organization and community.”

#### Tracking of Reported Leadership Activities

Of the 83 leadership activites reported, thirty-one (37.3%) directly referenced an EDI issue (Table 5). These activities largely show participants addressing changes in community and systems, either by addressing systemic issues that advance health equity (e.g., historic racism) or by being recognized for their work with communities. Participants reported systemic and institutional influence through their advocacy work, career advancements in large institutions and professional societies, and contributing to state and health data reporting policy changes.

**Table 5:** Cohort 1 Reported Post-graduation Leadership Activities by Activity Type

Leadership Activity Type and Description	
*Advocacy* (*n*=4)	
Participant interviewed on an episode of a podcast series about historical racism and its current impacts.	
Participant led the expansion of primary care, health screenings, and COVID-19 testing to medically underserved populations in their city.	
Participant invited to speak at a [national popular publication]-sponsored event about disparities and inequities in oral healthcare.	
Participant played a large role in convincing the leadership of a large medical professional society to sign on to a letter sponsored by the Asian Pacific Islander Health Forum and [academic medical center] Center for Asian American health to advocate for including Asian Americans, Native Hawaiians, and Pacific Islanders in the COVID-19 vaccine allocation plan by the National Academies of Science, Engineering and Medicine.	
*Award* (*n*=3)	
Participant named as one of [an influential social magazine’s] “100 women we love” for the work she does ensuring underserved populations have access to healthcare.	
Participant honored with an award from their professional organization for making outstanding contributions to their community.	
Participant received an award from [a national medical society] that honors practicing physicians who have made an outstanding contribution to the community for citizenship and public service.	
*Career advancement* (*n*=3)	
Participant announced to be part of the Health Subcommittee on their state Governor’s Council for Racial Justice. The group is tasked with counseling and monitoring the state administration in an effort to end systematic racism and promote equal treatment and opportunity.	
Participant invited to join a state university’s School of Social Work Community Advisory Council	
Participant appointed President of the Board for the [national advocacy organization].	
*Change in policy* (*n*=2)	
Participant contributed to the passing of a state bill, which prohibits discrimination against immigrants in local health benefits.	
Participant helped adopt and include race in reporting for Healthcare Effectiveness Data and Information Set measures for maternal mental health.	
*News recognition* (*n*=3)	
Participant featured in a [national medical society] article encouraging pediatricians for community engagement on various societal issues.	
Participant featured and interviewed in an article on the future of healthcare systems and the need for a more open impact within communities.	
Participant featured in a local news article about their work with community professionals to help push for better access to healthcare.	
*Program expansion* (*n*=3)	
Participant coordinated a food drive in their local community.	
Participants launched a new mobile dental unit to provide care at [early childhood] programs and in multiple low-wealth communities.	
CS project expanded an after-school program for a marginalized community group across their state.	
*Training* (*n*=10)	
Participant co-presented in a webinar series on the experiences of resistance and abolitionism in response to the colonization and policing of People of Color and Indigenous communities; Queer and Trans; and peoplewith disabilities in the Medical Industrial Complex.	
Participant delivered the keynote address at a global medical provider conference, describing approaches to heal the wounds created by the history of systemic racism in the field of medicine.	
Participant presented at an integrative medicine annual conference about lessons they learned about building power with communities and undoing harm through their community-based non-profit health clinic.	
Participant partnered with CS participants in other cohorts to deliver an interprofessional lecture on health inequities in the elderly to a graduate level public health class at a leading state university.	
Participant gave a talk on racism and health to the entire first year medical school class of a large state university.	
Participant led a digital panel discussion about healing historical injustices.	
Participant presented at a local event to facilitate community dialogue about public health and education.	
Participant was part of a panel at a local workshop about enhancing community-based harm reduction and treatment services in local communities	
Participant presented a webinar mini-series about the equitable distribution of the COVID-19 vaccine.	
Participant coordinated a free [early childhood parenting] program to be offered to a marginalized community.	
*Writing* (*n*=3)	
Participants co-published research on evaluation of development of a rural center of excellence in transgender health.	
Participant co-authored a study in a peer-reviewed journal about self-injury in transgender and gender-expansive youth.	
Participant co-authored an article in a peer-reviewed journal about preventative healthcare utilization by youths who have run away, experienced homelessness, or are stably housed.	

## DISCUSSION

Despite arriving with a high level of EDI skills, CS participants made significant gains in competencies related to equity, diversity, and inclusion. Specifically, this mixed-methods, multilevel evaluation demonstrated significant growth in organizational, community engagement, and system change competencies with participants reporting behavioral examples and leadership activities demonstrating application and results in organizational and system change.

“Boundary Spanning Leadership” is the ability to span vertical, horizontal, stakeholder, demographic, and geographic boundaries in service of a higher goal.^28–33^ Recently, the de Beaumont Foundation identified eight domains needed to address our current complexity: systems thinking, change management, persuasive communication, data analytics, problem solving, diversity and inclusion, resource management, and policy engagement.^34^ The field of leadership development has acknowledged we need skilled leaders to develop new solutions that better match the volatile, uncertain, complex, and ambiguous nature of our current society and that embrace the higher goal of advancing health equity. Our findings extend the leadership literature through providing program, competency, and curriculum descriptions for a comprehensive, robust program that demonstrates the ability of participants to acquire the skills to advance health equity, and to apply those skills to achieve organizational and system change.

The high level of proficiency in EDI skills of participants entering the program is likely reflective of both evolving societal understanding and appreciation for EDI and the program application requiring explicit articulation of a project to advance equity in a complex area in health. While strong capabilities in the personal, interpersonal, organizational, and system domains are important for all leaders, our finding of greater gains and impacts at the organizational and system levels that stem from our equity-centered framework shows that community engagement and system change competencies are crucial for healthcare system leaders to advance health equity. In addition, we acknowledge this type of program is unique in leadership development programs in both intensity and duration. However, the history of health inequities is long standing and efforts to this point have been insufficient in mitigating centuries of health disparities. We believe to move the needle in advancing health equity new frameworks and deep commitment, such as this program, are needed. While participants in our program on average entered with high levels of EDI interest, we did see heterogeneity in initial skills as would be expected, given that this journey both is quite personal and has no defined endpoint. We found that “buy-in” to these concepts varies as well, given that some individuals agreed *in principle* but were met with some realizations of their own bias or lack of awareness through the discovery process of the work. The curriculum was designed with this flexibility in mind and was able to alter the pace when greater processing was needed and capitalize on group support to help individuals continue moving forward on their journey. The intentional interweaving of evidence-based curriculum with flexible application exercises allows intensive leadership development programs such as this to be accessible to a wide range of audiences. As organizations continue to make pledges of institutional commitment to the values of equity, diversity, and inclusion, they need to develop and invest in leaders who can affect the changes embodied in their statements. Leadership journeys can begin with personal or interpersonal level skills, but leadership development programs can and should build on this foundation to ensure leaders have skills in organizational, community engagement, and system change critical to advancing health equity. Still, these leaders will require institutional endorsement.

This work adds to the literature in distinguishing between leader development and leadership development. Van Velsor, McCauley, and Ruderman^35^ define *leader* development as expanding the capacity of the *individual*, and “…*leadership* development as the expansion of a collective’s capacity to produce direction, alignment, and commitment.” CS offers both by including developmental learning experiences that increase both individual and collective capacity. Grimm et al.^36^ described the essential difference between developing leaders as a group of individuals and developing leadership. In developing, refining, and validating^6^ our equity-centered leadership approach, we have formulated ways to achieve both leader development and fostering leadership. Data here and across our dissemination of findings^5,6,34,35,37–41^ illustrate individual growth of participants and provide clear evidence of impact in organizations and communities alike as they function both as individuals and in teams to effect real and meaningful change. In the goal of advancing equity, individual development as a leader is a critical foundation and precursor to the form of collective, communal, and collaborative leadership that advances diversity, equity, and inclusion in organizations and communities.

As in all research, our study has limitations. Our design does not provide a control group and we were not able to assess the gains in participants compared with those who did not have the opportunity to participate in CS. However, our robust evaluation uses both qualitative and quantitative data triangulated across multiple levels of evaluation, showing consistent results that add to the confidence in our findings. We also need to acknowledge potential selection bias. In CS selection criteria, we looked for individuals who were ready to develop skills to advance health equity. The high self-reported levels of EDI skills at baseline suggest our selection efforts were successful in reaching the intended audience. When assessing the impact of the program through the most significant change approach and in leadership activities, we intentionally avoided asking participants specific questions related to EDI to keep open the full range of submissions. If we had queried about specific EDI-related impacts, the data would likely have been even richer and more compelling with regard to the impact program participants are having in their respective systems. However, the approach used allowed participants to define what was most salient for them in their experience. Finally, our evaluation was not able to identify whether there were specific components of the training program that resulted in the greatest learning gains in participants.

Leadership development will be critical to health-related organizations that seek to go beyond pledges of support for EDI. Our experience with an intensive leadership program that centers EDI skill development across personal, interpersonal, organizational, and systems domains demonstrates the potential to develop leaders who can create and sustain environments that advance health equity. The results presented here demonstrate healthcare professionals can gain significant and relevant skills to move beyond the level of self-growth and apply their newly acquired skills to impact organizations and communities in meaningful ways that advance EDI. The challenges before our nation are immense, yet our healthcare workforce is vast. Since healthcare professionals are trusted members of their communities, providing those professionals with equity-centered leadership models as a part of their career development could create significant and lasting effects for their institutions and communities and broaden the field of allies in the fight for health equity.

## Supplementary Information

ESM 1 (DOCX 35 kb)
