# Supplementary material for: Advancing Health Equity Through Equity-Centered Leadership Development with Interprofessional Healthcare Teams
Source: J Gen Intern Med. 2022 Jun 3;37(16):4120–9. doi: 10.1007/s11606-022-07529-x (PMC9165542; doi:10.1007/s11606-022-07529-x)
Supplement: Supplementary file 1 — (DOCX 35 kb) [file 11606_2022_7529_MOESM1_ESM.docx]

| *Appendix 1: Wilcoxon Signed Rank Test Results by EDI Competency, by domain, Cohort 2016* | | |
| --- | --- | --- |
|  | **Z-score^*^** | **P-value (**†**,** ‡**,** §**)** |
| ***Competency*** | | |
| Commitment to Intercultural  Development | -3.40 | .001‡ |
| Social Justice | -3.07 | .002‡ |
| Practice of Multi-  Culturalism | -3.39 | .001‡ |
| Organizational Capacity for  Health Equity | -3.46 | .001‡ |
| Diversity and Inclusion | -3.34 | .001‡ |
| Health Equity | -3.11 | .002‡ |
| Meaningful Community  Engagement | -3.33 | .001‡ |
| Social Determinants of  Health | -2.82 | .005‡ |
| ***Domain*** | | |
| Personal | -3.92 | .000§ |
| Interpersonal | -3.72 | .000§ |
| Organizational | -3.13 | .002‡ |
| Community & Systems | -3.40 | .001** |

*Based on negative ranks

† p<.05

‡ p<.01

§ p<.001
